# The AI Act in a law enforcement context: The case of automatic speech recognition for transcribing investigative interviews

**DOI:** 10.1016/j.fsisyn.2024.100563

**Published:** 2024-12-05

**Authors:** Radina Stoykova, Kyle Porter, Thomas Beka

**Affiliations:** a University of Groningen, Broerstraat 5, 9712 CP Grongingen, Netherlands; b Norwegian University of Science and Technology, Teknologivegen 22, 2815 Gjøvik, Norway; c Norwegian Police IT-unit, Fridtjof Nansens vei 14, 0031 Oslo, Norway

**Keywords:** Investigative interviews, Artificial Intelligence, Automatic speech recognition, Artificial Intelligence Act (AI Act), General-purpose AI models, Law enforcement

## Abstract

Law enforcement agencies manually transcribe thousands of investigative interviews per year in relation to different crimes. In order to automate and improve efficiency in the transcription of such interviews, applied research explores artificial intelligence models, including Automatic Speech Recognition (ASR) and Natural Language Processing. While AI models can improve efficiency in criminal investigations, their successful implementation requires evaluation of legal and technical risks.

This paper explores the legal and technical challenges of applying ASR models to investigative interviews in the context of the European Union Artificial Intelligence Act (AIA). The AIA provisions are discussed in the view of domain specific studies for interviews in the Norwegian police, best practices, and empirical analyses in speech recognition in order to provide law enforcement with a practical code of conduct on the techno-legal requirements for the adoption of such models in their work and potential grey areas for further research.

## 1 Introduction

An investigative interview report is a summary or a full transcription of the interaction between the police and the interviewee during an interview [1] “that can contribute to investigation and determination of the prosecution” [2]. Such reports are important as they “are often central to assessments and decisions, and may be presented to prosecutors, defense lawyers, and the court several times” [2] in the course of the criminal proceedings.

According to the Norwegian Attorney General [1], the prosecuting authority is guided by the principle that interviews should be conducted correctly, and “confessions and other particularly important statements” should be reproduced in the interviewees’ own words. Transcriptions can be summaries of the interview or more extensive depending on the type of crime, but in all cases the interviewee must confirm and sign the final text. Further, all investigative interviews conducted with vulnerable people or children under 18 years old [3] must be transcribed in full [4] with speaker diarisation.

A problem noted by Norwegian law enforcement agencies (LEA) is that the manual transcription of the investigative interview reports is a time consuming and tedious process. The use of Artificial Intelligence (AI) has been considered as a means to automate the process, thus potentially saving many valuable man-hours of work [5]. While a professional transcriber can transcribe one hour of audio in approximately 11 to 14 h,^1^ studies show that an automated transcription with human-in-the-loop verification can significantly improve the overall speed of transcription. This technology is generally referred to as automatic speech recognition (ASR), or speech-to-text.

In terms of state-of-the-art commercial and open-source tools, Wollin et al. [6] showed that it is possible to transcribe 3 min of audio in a minute or less with sufficient hardware resources. The study also showed that where an experienced transcriber could transcribe the three minute audio in roughly 10.5 to 11 min, the output of the best ASR system (Whisper Version 2) took between 3.5 and 6.5 min to correct (depending on the language). Older studies have also shown a potential in overall transcription time as well. The United States Army demonstrated that an off-the-shelf speech recognition system could transcribe a 15 min audio file in 1 min with over 90% accuracy, whereas the manual transcription took 32.5 min [7]. Anecdotally, we are able to transcribe a 27 min .mp3 file with Whisper Version 3 in 45 s with very high transcription quality using a single Nvidia A-100 GPU. In addition, ASR transcriptions have measurable and testable accuracy, bias, and error that can be verified by a human which can potentially reduce contamination of verbal evidence.

However, before adopting ASR models for transcribing police interviews it should be ensured that they do not introduce any negative effects to standards of criminal procedure, data protection, and AI regulation. Traditionally, criminal procedure requires accuracy and chain of custody for investigative interviews, while ASR models work with statistical language approximation, their inner workings are not fully traceable or verifiable, and certain outputs cannot be fully explained. Consequently, the automatic transcription of such reports must be complemented with standards for reliability assurance. This requires validation that the machine learning model works as intended and reproduces everything said in the interview without any biases or adverse effects in the transcription. In order to mitigate societal and human rights risks arising from AI systems the European Union adopted the Artificial Intelligence Act, [8] which entered into force on 1 August 2024. It is the first comprehensive regulatory framework for AI systems in Europe, including their use for law enforcement purposes. As it provides a general accountability regime, its application in the law enforcement sector with respect to different AI models requires further research and standards development.

Therefore, we focus on the following research questions:

• What is the legal classification of ASR systems for investigative interviews and the related LEA obligations according to the AIA?
• What are the challenges and mitigation strategies that law enforcement should consider in developing a voluntary code of conduct for ASR systems transcribing investigative interviews according to Art. 95 AIA?

The paper takes a first look at the technical and legal questions arising specifically from state-of-the-art speech-to-text AI models applied for investigative interviews. The consideration of ASR system compliance with the AIA is developed by using relevant AIA provisions to derive concrete requirements for ASR systems and a survey of relevant research papers to further concertise them in law enforcement work. We examine the practical challenges in meeting those requirements as well as possible mitigations or solutions.

We limit our examination of ASR systems to open-source models due to the fact that they are available for study and typically have a sufficient amount of documentation. In particular, we focus on two of the most popular and successful open-source ASR solutions, Whisper [9] and Wav2Vec2 [10].

The novel contributions of this study are as follows:

• We provide an argument that according to the AIA, ASR systems used for the purpose of transcribing investigative interviews are classified as minimal-risk systems and do not fall under the specific transparency obligation for general purpose AI (GPAI).
• The paper identifies a regulatory gap in relation to fine-tuning and repurposing of commercial AI models for law enforcement purposes, stemming from the different regime in AIA for providers and deployers of AI systems.
• The analysis outlines initial criteria to develop a voluntary code of conduct for ASR systems in accordance with Art. 95 AIA. According to the EU Commission, voluntary codes of conduct are meant to advance AI literacy among persons dealing with the development, operation and use of AI systems in different domains. Our in-depth analysis explores the existing practical challenges for ASR systems voluntary code of conduct according to the AIA high-risk requirements. The AIA provisions are interpreted and extended with best practices for legal compliance, standard metrics, and empirical ASR studies to support law enforcement in the assessment of ASR systems and to identify potential pitfalls, mitigation strategies, as well as gaps in the research. The provided initial voluntary code of conduct may be used to analyse other AI systems in an investigative context as well.

The structure of the paper is as follows: Section 1 stated the purpose of the paper. In Section 2 we provide a background on ASR models, basic details regarding the two discussed open-source models, and an overview of similar and related work. In Section 3 we discuss ASR risk classification and some challenges emerging from the use, fine-tuning, and repurposing of general-purpose, commercial, and open-source ASR models for law enforcement tasks. In Section 4 we examine ASR applied to investigative interviews in relation to each provision of AIA in order to highlight possible compliance propositions, challenges, and mitigation strategies. In Section 5 we answer our research questions, highlight the main identified challenges and mitigation measures, and introduce the suggestions for a voluntary code of conduct (in Table A.1 in the Appendix) for ASR systems in order to assist law enforcement in the evaluation of such models and bringing them in compliance with AIA. The section also discusses the limitations of our study, while in Section 6 we provide final remarks and thoughts on future work.

## 2 Background and related work

This section gives a brief introduction of state-of-the-art ASR models, performance metrics, and their previous applications in legal contexts.

### 2.1 Automatic speech recognition

The research problem of automatic speech recognition can be traditionally boiled down to designing a model that attempts to find the most likely transcription given some auditory input. Recent developments for speech to text models have focused on the use of deep learning models with so called “end-to-end” ASR architectures, wherein a single deep neural network is used to take feature vectors of audio as input, and to learn and output the transcription of the audio directly [11].

We now briefly introduce two of the state-of-the-art open-source ASR models which have been surpassing performance benchmarks within the field of ASR. These models can be considered as “foundation models” models, as they were trained on massive pre-training datasets and can be used to train for a specific language transcription task, or they can be used outright for transcription of a broad set of languages (see Section 3.1.1 for further discussion). We describe their capabilities here as it is relevant to understand the compliance challenges in Section 4.

#### 2.1.1 Wav2Vec2

Wav2Vec2 [10] (and its subsequent and more robust versions [12]) uses a transformer model and training algorithm that is motivated by the fact that most languages are “low-resource” languages, meaning there is very little training data available to train models in that particular language. Training data typically requires the sampled data, such as the spoken audio files, and then the *labels* for the given data, such as the transcription of the spoken audio. Especially in the field of deep learning, this “pre-training” dataset is required to be massive if the desired model is to be of any use. In order to bypass the requirement of a large supervised (labelled) dataset, Wav2Vec2 utilises *self-supervised learning*. Essentially, this means that the training algorithm for Wav2Vec2 hides part of the audio input and must predict the values of the hidden sounds based on the surrounding context. Users of the pre-trained model need only one hour or less of labelled and domain-specific training data (such as audio-text pairs for a specific language or business domain) to further train (*fine-tune*) the model in order to see relatively useful results.

Ultimately, the trained model maps the audio input to particular learned discrete speech units. These speech units are akin to phonemes in human language, which are the smallest units of sound in human speech. This then allows the model to spell out the audio without the need of a language model (a probabilistic model showing the likelihood of the next word following the proceeding words). However, Baevski et al. [10] show that they can improve their results by including the use of an external language model in combination with the end-to-end model.

#### 2.1.2 Whisper

OpenAI’s Whisper [9] argues that the self-supervised nature of Wav2Vec2 prevents it from being used “out-of-the-box”, and that fine-tuning is still a complex process and the major component of mapping speech audio to text. Thus, Whisper is pre-trained on an unprecedented amount of multilingual data with audio to text pairs, so the user does not necessarily need to fine-tune the model. What makes their model special, other than the training data, is that it utilises *weakly-supervised learning*. A benefit of weakly-supervised learning is that the audio transcription can be imperfect, such as video subtitles or meeting transcripts. The model is considered a “sequence-to-sequence” model, in that it essentially learns mappings between sequences of audio to sequences of text.

The most recent updates to Whisper Version 3 have been pre-trained with weakly-supervised learning on even more data than before (one million hours), and additionally trained with four million hours of pseudo-labelled data (data labelled by Whisper Version 2 and then use of weakly-supervised learning). The authors claim that errors of the newer model have been reduced by 10%–20%.^2^

#### 2.1.3 Error rates metrics

ASR systems typically benchmark their performance using the *Word Error Rate* (WER) [13]. This is essentially the edit distance presented as a percentage between a reference text, and another transcribed text. The edit distance is calculated as the total number of errors between the reference text and the machine transcribed text, where possible errors are word insertions i, word deletions d, and word substitutions s. The sum of these errors are then divided by the total number of words n found in the reference text. The following equation writes out the word error rate explicitly:

$$\text{WER}=\frac{i+d+s}{n}$$

Other possible metrics can be used for ASR, such as the Character Error Rate (CER) which counts the difference between different characters rather than words, as well as using semantic similarity to compare transcriptions [14].

### 2.2 Studies of ASR use in legal contexts

Past research has considered the use of ASR within legal contexts, where findings have either focused on the performance results within their specific domains, or the possible implications or consequences of using such technology.

Harrington [15] conducted perhaps the only research applying ASR in the context of police investigative interviews, where “mock interviews” were used as the source of analysis. The purpose of the study was to compare three different commercial ASR tools (Amazon, Rev, Google) on how they performed in terms of types of errors produced (insertion, deletion, substitution), regional accents, and the effect of added synthetic noise. The primary results found that there was no statistical significance in the difference between accents, but the possible distribution of errors in non-standard accents may be more problematic when attempting to correct interview transcripts. The study however did not include proper nouns, nor did it comment on the domain of the investigative interview and the possible effect of their vocabularies. Their research also emphasises the need to further explore the actual timing benefits in using ASR supported transcriptions over traditional human written transcriptions.

In the more general field of forensics and investigation, Negrão and Domingues [16] created a third party ASR module for the open-source digital forensics suite, Autopsy, wherein they could enable the automatic transcription of sound or video files. Using open-source ASR and Voice Activity Detection engines, they were capable of identifying all files with human speakers, with Word Error Rates of 27.2% for non-native English speakers and 7.8% for the clean Librispeech test dataset [17]. Vásquez-Correa and Álvarez Muniain [18] compared the performance of Whisper and Wav2Vec2 models across multiple languages within the domain of child exploitation, where relevant data was extracted from open datasets, and then performed keyword spotting on the automatic transcriptions. Their results showed that Whisper performed best in the majority of datasets and languages for both transcription and keyword spotting (especially in cases where there were “uncontrolled acoustic conditions”).

Loakes has conducted multiple studies considering the application of ASR for the transcription of “indistinct” covert recordings, as a means to prove what was being said in the recording [19], [20]. Her findings concluded that, despite recent advancements in ASR models, the current state of the art was not reliable enough to solve the task. Wahler [21] considered the implications of relying on translation AI within the American justice system, stating that such technology should be seen as “an imperfect assistant whose work will always require subsequent review.”

Finally, and most relevant to our paper, a study by Lorch et al. [22] discusses the general provisions in AIA and their interpretation in forensic image analysis for law enforcement. The study outlines several challenges with the legislative regime related to specifics in licence plate image recognition and shows that contextualising AIA requirements in specific law enforcement domains provides valuable insights on limitations and open research areas for AI-based image analysis systems. Our study focuses on domain-specific evaluation of ASR models in the context of AIA as well, but it is not constrained to a general literature review of challenges. Rather we discuss best practices and empirical studies which can give a direction for in-depth evaluation and research of ASR systems according to the AIA framework for pre- and post-model development management.

## 3 ASR risk classification and general compliance obligations

This section provides an assessment of the legal regime for ASR models in law enforcement work by examining the risk classification for AI systems in the new AIA and the specific transparency and explainability regime for general purpose AI models (GPAI). The role of law enforcement in AI systems development and use is also discussed in order to clarify their obligations for compliance with AIA.

### 3.1 ASR models risk classification

The AIA adopts a risk-based approach to AI systems regulation. This means that some AI systems are prohibited, while all others are classified in different risk levels. AI systems which pose greater risks to society, safety, and human rights must comply with stricter rules (Recital 14 AIA). Firstly, ASR systems for investigative interviews do not fall under any of the prohibited AI systems that are explicitly listed in Art. 5 AIA. Therefore, it should be examined if such systems are regulated under:

• high-risk (subject to strict conformity assessment),
• limited risk (subject to transparency obligations),
• or minimal risk (no obligations but voluntary compliance encouraged).

High-risk AI systems in law enforcement work such as biometrics identification, risk assessment or profiling are exhaustively listed in Annex III AIA, but ASR models do not qualify as such systems. In general, processing the voice of a person is biometric data if it is used to identify or categorise people. However, ASR systems do not have any such capabilities and therefore does not fall under high-risk AI systems for biometrics in Annex III (1). The ASR transcription systems are intended to improve the result of a previously completed human activity in law enforcement work, i.e. converting the interview data from its audio or video form to its text form without altering the information. Although, transcriptions of investigative interviews might be used as evidence in court (e.g., witness in absentia or dead), ASR models do not affect the actual administration of justice or the interpretation of the facts by the judge so they will not fall under the Annex III (8) definition. As the AI system performs purely administrative assistance and cannot be used for assessing the reliability of the verbal evidence, they do not fall under the scope of Annex III (6)(d) AIA either.

ASR models are not used for any of the purposes requiring transparency obligations in Title IV such as artificially generated content, emotional recognition or biometric categorisation. As examined further in Section 4, ASR systems for police interviews do not pose significant risk for the fundamental rights, as long as an ASR system provides only a draft transcript that is further verified by a human transcriber and by the interviewee.

Consequently, it can be concluded that such systems will be classified as minimal risk for which the AIA does not provide any specific obligations. Minimal risk AI systems can be however subject to specific transparency requirements if they implement general purpose AI (GPAI) models as examined further.

#### 3.1.1 Are ASR models for law enforcement GPAI models?

The AIA strictly distinguishes between AI systems and GPAI models, as the latter are considered under a separate regulatory regime that is applied cumulatively to the risk-based assessment. A complete AI system encompasses a user interface, the input data, and the algorithms that learn to identify patterns and correlations from such data (Art. 3 (1)). According to Art 3 (63), an AI model is general purpose if: (1) it is trained with a large amount of data (2) uses self-supervision at scale (3) displays significant generality and (4) is capable of competently performing a wide range of distinct tasks. Although AI models can be essential components of AI systems, they do not constitute AI systems on their own (Recital 97).

Two types of AI currently can fall under the definition of GPAI model-generative AI and other types of foundation models. Generative AI refers to deep learning models are capable of generating content like text, video, images or code depending on the provided input. On the other hand, language models like BERT are not generative models, but much like DALL-E and GPT-3 are described as a “paradigm shift in AI” as they are “trained on broad data (generally using self-supervision at scale) that can be adapted to a wide range of downstream tasks” [23]. Such models are called foundation models to underscore their critically central yet incomplete character. Thus, it is relevant to ask if ASR foundation models pretrained on massive datasets^3^ to enable more general use and subsequent fine-tuning fall under the legal definition of GPAI.

Whisper and Wav2Vec2 may satisfy the first GPAI condition, in that both models have versions that are trained on hundreds of thousands of hours of speech data [9], [12]. However, only Wav2Vec2 satisfies the use of self-supervision pretraining at scale, and even then, there are significant caveats. Wav2Vec2 only learns general acoustic speech patterns from this data, but must be additionally fine-tuned with a relatively small supervised (labelled) dataset to effectively transcribe. On the other hand, Whisper models are pretrained using weakly supervised learning. Thus, for transcription the models are only learning the mappings between audio and text data, even if the labelled data (the transcriptions) has not undergone human verification. Furthermore, the more recent versions of Whisper utilise pseudo-supervised learning, where training labels are provided by previous versions of Whisper. The AIA provision refers only to self-supervision and it should be clarified if this should be interpreted restrictively or broadly to encompass middle way solutions such as pseudo- or weakly-supervised learning. As both of those methods involve limited human intervention in terms of labelling, we can assume that a broader interpretation of the provision to include them as well is preferable.

Thus, both Whisper and Wav2Vec2 only partially fulfil the second requirement to be considered GPAI, it is questionable if they meet the third and fourth conditions regarding generality and their ability to perform a wide range of *distinct* tasks. The third requirement is that the model should have generality determined by the number of parameters where “models with at least a billion of parameters and trained with a large amount of data using self-supervision at scale should be considered to display significant generality and to competently perform a wide range of distinctive tasks” (Recital 98). The legal concept of generality however is quite unclear. It seems that there is a legal assumption that “generality” and the fourth requirement “capability to perform wide range of tasks” are necessarily conjointly present. However, the fact that a model has generality does not mean that it has capabilities to perform wide variety of task.

Both Whisper and Wav2Vec2 have versions of their models which fall beneath and above the one billion parameter threshold, but according to our interpretation, the models do not meet the fourth criteria of being capable of accomplishing a wide range of distinct tasks due to the nature of their pretraining. Though using self-supervision, Wav2Vec2 limits the model to learn a set of “discrete speech units” for the foundation model [24]. Whisper, using weakly-supervised learning, is primarily pretrained to perform one task — mapping segments of audio to segments of text. Researchers also proposed a narrower understanding of task noting that: “Although systems may be coupled or combined with others to complete a higher number of tasks, if each of them is limited to performing the task they are originally trained for, they qualify as fixed-purpose” while GPAI can perform variety of tasks, including some that “they were not originally trained for” [25]. For example, GPT models trained for next word prediction led to a model, using a massive number of trainable parameters, with many emergent capabilities such as summarisation, translation, question and answering, etc. By contrast, Whisper undergoes language detection and translation in the pretraining [9], but cannot be used to perform a “variety” of distinctive tasks they were not explicitly trained for like ChatGPT. As both Whisper and Wav2vec are suitable only for limited tasks related to transcription, we can conclude that ASR models for transcribing investigative interviews are minimal risk AI systems that do not implement GPAI models.

However, according to Art. 95 AIA even providers of minimal risk AI systems are encouraged to voluntarily assess their systems for compliance with the requirements for high-risk AI, in order to improve overall literacy and transparency of AI systems. Voluntary assessment of the ASR system for investigative interviews will be most beneficial considering that any automation introduced in criminal procedure must be carefully examined for potential negative impacts on the rights of individuals. Before we conduct such examination however, we need to clarify what is the LEA role in the development and use of ASR systems for investigative interviews as the AIA provides different compliance obligations for providers and deployers of AI systems.

### 3.2 Are law enforcement providers or deployers of ASR systems?

In this section, we analyse in which situations law enforcement are considered providers or deployers of AI systems and models in order to exemplify challenges in public–private relations that can create obstacles to identify who should perform an assessment of the AI system. As compliance obligations are defined only in a high-risk context, we apply this logic by analogy to ASR low-risk systems as we aim at a voluntarily compliance with AIA.

The AIA defines two roles: *Providers* are the developers of the AI system, those who put it into service (Art. 3(3)) and in case of high-risk systems they must comply with all AIA requirements (Chapter III), while *Deployers* are only using the system under their authority and have limited compliance obligations (Art. 3 (4) and Art.26). AIA imposes most responsibilities for the design and safe implementation of AI systems on the providers, while deployers must perform fundamental rights impact assessments, use the system safely, and ensure human oversight for its specific use (Art. 26 and 27 AIA).

Ebers et al. [26] argue that this role distinction is problematic for *(i)* general-purpose AI systems that can be used for many different purposes (some unforeseen by the provider/developer); *(ii)* identifying users‘ responsibilities in AI as a service (AIaaS) or *(iii)* responsibility for post-deployment operations by both developers and users. We support this argument and analyse it further to show that it is particularly problematic in law enforcement contexts. Due to the specificity of this domain it might become increasingly difficult to decide who is a provider of an AI system, while the role separation between provider and deployer could be considered too simplistic in practice due to fine-tuning or repurposing of AI systems or commercial models for law enforcement tasks.

#### 3.2.1 Law enforcement and commercial providers of AI systems

One plausible scenario is that law enforcement might decide to purchase commercial ASR tools or AI as a Service (AIaaS) solutions with built-in AI features. In this case law enforcement will be deployers of the tool while the commercial vendor is the provider. This scenario might already be problematic as the tendency of LEA to use all-in-one tools, that they them-self have not tested, creates technology dependencies and reliability issues in LEA work which are well studied outside the AI context [27], [28], [29]. Most notably, law enforcement have an obligation to assess the reliability of the evidence they adduce in criminal proceedings, which require validation of AI tools output [30], [31]. Further, if LEA are just AI system deployers, then the status of AIaaS and commercial AI tools providers must be clarified in the view of the specifics of criminal procedure. For example, it is undesirable for private tool providers to be in a position to evaluate fair trial and discrimination risks in criminal justice context. Finally, commercial AIaaS and AI tools for law enforcement purposes can hardly be validated post-development by the commercial provider since LEA will most likely insist on controlling their police datasets and not disclose them to the AI developer. In such cases, the AI developer is ill-equipped, for example, for post-development tests for quality assurance and continuous monitoring due to lack of access to specific police data, while LEAs as deployers have only limited obligations for post-development monitoring.

Another scenario is when the police have a dedicated developer team to serve as an AI system provider, while all other law enforcement units are just AI system deployers. This scenario raises questions on enforcement of compliance and liability within the same organisation. Such issues might require reconsideration of the obligation of providers and deployers in law enforcement contexts.

Further guidance is required in terms of obligations on commercial providers of law enforcement AI tools. In particular, special attention should be given to the question posed by Crawford: “should private vendors be legally accountable for the harms produced when governments use their systems” [32, p.199]. Arguably, private vendors who provide AI systems for domains like law enforcement should be subject to separate independent validation regime and stricter requirements, even for minimal risk tools, as such tools should meet the high-quality standards of criminal procedure.

#### 3.2.2 The issue of fine-tuning ASR models on police data

Another set of issues arise if law enforcement decides to be a provider and to develop an ASR system themselves. It is unlikely that they will develop the system from scratch on a larger scale. The most plausible scenarios are that they will use: *(i)* foundation open-source or commercial models that have been trained for general language transcription; *(ii)* foundation models that have been fine-tuned for improving a specific language, or *(iii)* fine-tune foundation models for law enforcement tasks. In terms of high-risk systems these scenarios are resolved through Art. 25 AIA. The provision stipulates that the actors in the AI value chain are to be considered providers in the case they repurposed or substantially changed the AI system. In such cases, the initial provider must cooperate closely with the new provider to comply with AIA, but only if the initial provider allows repurposing of the AI system for high-risk use. It is questionable, if the GPAI provider or foundation model provider should be entitled to prohibit high-risk use of their model, in cases where law enforcement needs must be met. In addition, Art. 25 does not consider the repurposing or reuse of AI systems or models for non-high risk systems, while we demonstrated that some foundation models for ASR purposes might fall out of the scope of the GPAI definition. To avoid ambiguities, it should be explicitly interpreted that when minimal risk AI systems are repurposed and reused they also require the cooperation of the initial provider.

Another source of legal uncertainty is the question if fine-tuning of commercial ASR foundation models for investigative interviews qualifies as repurposing or substantially changing the model and therefore makes law enforcement providers of the newly-purposed system. In terms of high-risk systems, Recital 128 AIA clarifies that an AI system is considered new when there is a “change of operating system or software architecture or when the intended purpose of the system changes”. Fine-tuning does not introduce any changes in AI architecture and it could be argued that it does not change the purpose of ASR systems since they are still used for transcription. However, we would rather argue to the contrary. The ASR commercial system is used in a new public domain for law enforcement; it is also fine-tuned and tested on a very specific police datasets and for a new context namely investigative interviews. This leads to the conclusion that due to fine-tuning the ASR model is used for a new purpose, possibly not initially foreseen by the commercial providers.

In light of this interpretation, when LEA re-purposes a general or open-source model for their specific task, they should be considered providers of the newly purposed AI system. Thus, LEA must perform application-specific validation of the re-purposed AI model, which might also create challenges not resolved by the AIA. For example, LEA can account for risk management and testing of the system only in relation to the fine-tuning of the model and domain specific data testing. Though, open-source or commercial AI models might be accompanied with provider documentation for their general purpose and training, such information might not include law enforcement use. For example, datasets used for training commercial models are often not available for LEA and are not specifically designed for LEA purposes. Further, it is questionable if such complicated testing of repurposed models is economically and technically feasible for each law enforcement agency or a dedicated body should be established to assist LEAs.

In conclusion, the regime for providers and deployers is not considered in relation to public–private cooperation and creates dependencies for law enforcement on the initial AI providers which in the domain of criminal procedure are best to be avoided. In contrast to the strict regime on high-risk AI, the AIA does not provide any concrete obligations or clarification on the use of minimal risk systems. However, even minimal risk AI systems and non-general purpose models can create important legal issues when development and deployment of such systems is within a public–private dependence.

Further we consider law enforcement as a provider of ASR systems for interviews in order to outline the prerequisites for a voluntary code of conduct that can enable the police units to assess both commercial and in-house ASR models according to the AIA. Such a voluntary assessment is crucial to assess the impact of automated system on criminal procedure as the fact that a system is minimal risk does not exclude law enforcement responsibility to safeguard criminal procedure from negative effects of automation.

## 4 Prerequisites for a voluntary code of conduct for ASR systems in law enforcement

In this section, we discuss criteria to develop a voluntary code of conduct for ASR systems used for law enforcement purposes in accordance with Art. 95 AIA. Art. 95 AIA aims to foster AI literacy by encouraging providers of non-high-risk AI systems to adopt partially or fully and adhere voluntarily to the requirements for high-risk AI systems (as laid out in Title III). We consider the relevant AIA provisions in the context of ASR model development specifics, research studies, available technical solutions, and best practices in order to provide practical criteria for law enforcement to assess such models. Such voluntary assessment of ASR models for law enforcement tasks is considered necessary due to the novelty of such models and the need to study their potential negative effects in a domain of such sensitivity as criminal procedure. Therefore, we further examine challenges of voluntary compliance of ASR for investigative interviews with the AIA high-risk requirements, as well as possible methods to identify and mitigate them. Our analysis tends to take place from the Norwegian perspective as a case study, but the techno-legal findings are intended to be generalisable to all ASR models and languages which may be subject to the AIA.

### 4.1 AI risk-management system and fundamental rights impact assessment

Art. 9 AIA requires the AI system to have two components: *(i)* a continuous process to identify, analyse, and evaluate risks to fundamental rights of persons (Art. 9 (2-4) AIA) and *(ii)* a testing procedure to identify suitable risk mitigation measures. The risk management process and testing should be limited to the intended purpose, including the specific context and conditions of use, or foreseeable misuse of the AI system (Art. 9 (5-7 AIA) in conj. Art. 3 (13) AIA). Schuett [33] points out that the risk management process requires systematic use of available information to identify potential sources of harm while the greater the potential impact of the risk, the more effort an organisation needs to put into foreseeing and reducing it. However, NIST has emphasised that “measuring the risks in early stage of the development of the AI system might be different from later stages” or application-specific testing in real world conditions [34].

A preliminary literature review identified three main fundamental rights risks with ASR models for transcribing investigative interviews, where the relevant risk management processes and testing are examined further.

#### 4.1.1 Fair trial risks

Although investigative interviews are subject to jurisdiction-specific rules, they have some common characteristics as a way to obtain verbal leads. To ensure compliance with the fair trial principle, most countries implement specific safeguards in the methodology to perform the interview such as caution, legal advice, reasons for interview, and giving opportunity for the interviewee to clarify misunderstandings [35]. Police interviews have investigative significance and in exceptional cases can be used as evidence [36]. For example, although the Norwegian criminal procedure requires witnesses and victims to give statements in person on trial, there are circumstances when this is not possible and the transcriptions are used. Further, formal suspect interviews are likely to be adduced as evidence e.g. in case of obtained confessions or as a comparison material when the suspect changes her statements at trial, or at least as a decisive piece of information for the prosecution about the charges on the indictment. The actual piece of evidence is the audio recording, not the transcription, but in practice the transcriptions are used as signed copies of the original [37].

As a current means to ensure the accuracy of transcriptions in the Norwegian legal system, police-suspect interview reports should be read out for adoption, the defense should be given the opportunity to examine the transcription for inadmissible or prejudicial information and to request changes or omissions.^4^ Finally, both the interviewee and the person who wrote the report must sign it.

In order to assess the risks to fair trial imposed by ASR systems it will be beneficial to test the system against human performance for its ability to mitigate existing human-errors in order to improve the quality of verbal evidence. Human produced transcriptions are reported to contain omissions, inclusions, or distortions which can seriously impact the integrity of verbal evidence [38] due to poor performance by the transcriber, differences between spoken and written language, and transformations of the text that usually benefit the prosecution [37]. Another well-researched problem is that interviewee statements are given in concrete context and for a specific audience, while later they are re-narrated and re-contextualised repeatedly, which might lead to a complete distortion of the information presented to the judge [37]. Due to these human characteristics, ASR systems may benefit the criminal procedure by reducing human error in transcriptions as such systems predictably transcribe the speech of anyone despite how they may superficially appear. This can reduce issues like transcriptions skewed towards the hypothesis of the prosecution or initial subjective prejudice or stereotypes against certain people.

Studies show that ASR models experience inaccuracies as well (see Section 4.5.1) but they are quantifiable in testing, while in a semi-automated scenario where the transcription output from an ASR system is combined with a human listening to the file “may result in gains in both transcription time and accuracy” [7]. Akin to the standard investigative interview, a risk-management process should include a procedure to verify the integrity of the ASR-transcription and its accuracy via a human examiner while the interviewee should have the opportunity to object inaccuracies in concrete statements (see Section 4.4).

Such a verification should also account for the fact that sequence-to-sequence models such as Whisper have the problem to *hallucinate* statements that the speaker never said. It is common for models to produce misspellings or mishearings of particular words or phrases, but hallucinations differ by being often relatively lengthy text which are unfaithful to the original audio and have the possibility to be harmful [39] (see Section 4.5.2 for more details). While the occurrence of hallucinations may be relatively rare [39], their inclusion in an interview transcript may significantly alter what the interviewee intended to say. As hallucinations can be related to made up names or facts, they can potentially introduce inaccurate evidence, prejudice against the interviewee, and even impact the suspect‘s presumption of innocence. Therefore, we emphasise the importance of having a human-in-the-loop to verify any ASR produced transcription.

This brief overview, shows that fair trial risk mitigation in ASR systems is not only related to safeguards in the AI system, but also evaluation if the existing rights and obligations in the criminal procedure for police interviews are sufficient in the context of their automation.

#### 4.1.2 Data protection risks, requirements, and challenges

Law enforcement must comply with EU data protection rules in the Law Enforcement Directive (LED) [40]. Such compliance must be addressed separately and is out of scope here. It should be noted however, that processing of personal data with an ASR model is a new processing operation which requires a separate data protection impact assessment. Voice and speech are sources from which personal and/or biometric and/or sensitive data can be extracted but they themselves are not personal data [41]. By contrast, the speech recordings, voice templates and samples are protected by LED as personal data as they are linked to concrete individuals. Voice and speech data can be considered as a special category of data under Art. 10 LED as they might reveal health or emotional status (e.g., coughing, laughing, shaky voice); racial or ethnic origin (e.g., dialect or pronunciation); social status (e.g. slang). Therefore, the lawful processing of voice data shall be allowed only where strictly necessary, subject to appropriate safeguards for the rights and freedoms of the data subject and authorised by Union or Member State law (Art. 10 (a)).

Apart from the voice samples that might contain sensitive data, ASR systems will not typically transcribe sensitive non-verbal data into text if they were not included in the original training dataset. For example, Wav2Vec2 can be trained to capture generic kinds of nonverbal noises (for example, nasal cues), while Whisper models can be trained to capture complex annotations such as [laughter] or [inaudible] (which is possibly present in the pre-training data). Slang may or may not be transcribed in a standardised way, but this is dependent on the model being used. Models like Wav2Vec2 will likely output slang as it tends to “spell out” the transcription, whereas Whisper will likely output a standardised version of the word (for example, “going to” versus “gonna”). It is up to law enforcement to decide on a model which might allow such sensitive data to be transcribed. If voice samples or speech templates are processed for biometric identification, they will be considered biometric data (Art. 3(13) LED). Processing of voice data in police interviews and transcribing it to text does not constitute biometric-based voice recognition system in the sense of Art. 3 (13) LED provided that the AI system does not have the capability to uniquely identify a person.

Voice datasets must comply with safeguards for personal and sensitive data processing. For sensitive data it is recommended the consent to be written. The individual should be informed that an AI system is transcribing the interview and the related data subject rights to information, access, rectification and erasure of the transcription which stem from LED regime and criminal procedure as well.

#### 4.1.3 Non-discrimination risks and mitigation strategies

A well-discussed issue for ASR models is the performance gap that exists with respect to applying ASR to different demographics, in particular to minority groups and possible discrimination and reinforcement of societal stereotypes based on nationality, ethnicity, and gender. The cause of bias to ASR systems is typically attributed to imbalanced datasets but can also be introduced via training architecture and involved features [42], [43].

Bias risk mitigation requires AI providers and users to be able to identify if different demographic groups will be significantly and negatively affected by ASR solutions. Microsoft considers that for speech-to-text applications, particular demographics may suffer from quality-of-service harms, which can be measured by quantifying the difference between the calculated word error rates of different demographics [44]. Other researchers have delved further into taking quantitative approaches to measure fairness in the speech-to-text domain. Rajan et al. [45] created an automated tool to detect if ASR systems were equally robust across different demographics of speakers. This is done by taking audio samples from a demographic and applying perturbations to the audio and measuring the increase of errors between the “pure” sample and the perturbed samples. Thus, demographics that result in greater error rates illustrate that an ASR system is less robust for those demographics, and there is a quantitative quality-of-service difference. Liu et al. [46] and Markl [43] take more traditional approaches to distinguishing fairness across different demographics by employing statistical significance tests. Liu et al.’s statistical tests are derived from NIST’s approach for testing statistically significant improvements in ASR [47].

In terms of mitigation, previous work typically refers to having underrepresented demographics more present in the training dataset. This suggestion has been given by Koenecke et al. [42] and has been shown to work via oversampling underrepresented or poorly performing demographics (without reducing the overall recognition accuracy) [48]. Other mitigation methods include training on a more diverse training dataset (in terms of domain and quantity), which allows models to perform better in general on out-of-domain testing data [9], [49].

In summary, AI risk management systems for law enforcement purposes requires a holistic approach to risk assessment, which in addition to system requirements, considers procedural safeguards in the concrete criminal procedure of investigative interview ensuring that automation will not impact the quality of the procedure or the rights of the parties in the proceedings.

### 4.2 ASR data governance challenges

As performance of machine learning models, including ASR, depends on the quality of the datasets used, Art. 10 AIA defines concrete data management practices and stringent requirements for quality of datasets. An EU study reported that currently there are no universally agreed standards for data quality assessments for machine learning datasets [50]. The study concludes that such standards must be domain and AI technology specific. This paper extends those considerations in the domain of ASR models for law enforcement purposes.

#### 4.2.1 Data management practices

Art. 10 AIA requires data collection practices to be explicitly stated and include any design choices, assumptions, and biases examined in relation to the datasets. As argued, it remains unclear from a legal point of view, whether the police can use pre-trained foundation models from commercial or open-source vendors and if the police would have enough data to sufficiently fine-tune such models.

Each of the requirements in Art. 10 AIA is rather stringent and as shown below might bring compliance challenges in ASR model development.

**Data Preparation Requirements**

Art. 10 (2)(c) AIA requires data preparation operations, such as annotation, labelling, cleaning, enrichment and aggregation to be appropriately selected for the purpose of the model. For ASR models, this typically includes finding audio with a sufficiently low signal-to-noise ratio [9], [43], obtaining reliable transcriptions of the audio files, and pre-processing the audio files in a standard fashion. Aksënova et al. [51] suggest that data preparation includes consistent audio encoding formats, consistent audio sampling rates (typically 16 kHz), and consistent transcription conventions. For training models, it is also required that audio files are limited to sufficiently small segments of audio such that they can be ingested by a model (typically less than 30 s). This can be done manually, or use of voice activity detection tools such as Silero^5^ can support this activity.

**Data Preparation Challenges**

Challenges for compliance regarding governance practices such as data preparation is dependent if the provider of the foundation model has compliance obligations, if a down-stream provider has compliance obligations, or if the police themselves are considered to be providers or if they fine-tune any foundation models.

Article 10, Sec. 2 describes the obligations for testing, training, and validation datasets should subject to data governance practices that are suitable for the intended purpose of a high-risk AI system. Given that the provider of the foundation model, the down-stream provider of a language specific model, and a deployer all have different purposes of the model, the content and objectives of their datasets may differ extensively. Some of the first elements to consider for data governance compliance are the following: origin and availability of the data, its quantity, and its purpose and suitability for the system’s intended task.

Even amongst the providers of open-source foundation ASR models, some providers will have more difficulty demonstrating compliance than others. OpenAI’s Whisper Version 3 model [52], which was trained in a fashion that extends how Version 2 was trained [9], conducts its pre-training with one million hours of labelled data and an additional four million hours of pseudo-labelled data (data labelled by Whisper Version 2). The origin of the is unspecified, other than the fact that the models are trained from audio-text pairs found on the internet, and thus the data is not available for the purpose of verification. In terms of quantity, purpose, and suitability of the data for its purpose, the Whisper models are unprecedented when it comes to creating a robust foundation model intended for general speech to text tasks in a variety of languages, and the benchmarks of the models tend to support this. The authors also argue that the inclusion of sufficiently noisy, but well annotated, audio files make the model more robust.

The Wav2Vec2 model [10] (and its derivatives [12]) are far more open with regards to the origin of their pretraining data, and essentially do not have any compliance issues with respect to origin, availability, or suitability. Meta particularly specifies that they train with publicly available data, where the large multilingual Wav2Vec2 (XLS-R) model was pre-trained with 436 000 h of unannotated speech. The purpose of the model is to have a model that only requires a relatively small amount of data for fine-tuning to a specific language, and as the pretraining data covers 128 different languages, the choice quantity of the data appears suitable for its intended task as the datasets also contain both read and spontaneous speech.

The previously mentioned foundation models are intended for general use, where the majority of the data are pretrained on the English language. For instance, 65% of Whisper Version 2’s pretraining data is English, and only 17% of the data is “multilingual” [9]. Thus, other parties have produced datasets for the purpose of fine-tuning foundation models to better fit specific non-English Languages. Such *low resource languages* (including Norwegian) will simply not have the possible data sources that English has [53], and fine-tuning the existing pretrained models in their own language or dialect will be dependent upon efforts to explicitly produce a sufficient amount of audio-text pairs, especially while abiding data protection rules.^6^

Perhaps the greatest burden for compliance to demonstrate origin, availability, suitability for their purpose would be if the police themselves were to train or test models on real investigative interviews. In their case, the purpose of the AI system should be that transcription works sufficiently well for investigative interviews. Fine-tuning a foundation or otherwise downstream model for this task would require use of the police’s own internal data, where the recordings and transcriptions were not initially intended to be used for the purpose of training an AI model and of a sensitive nature. The suitability might be appropriate, but it also depends on the diversity and quantity of such data. Studies have shown that pre-trained ASR models fine-tuned with insufficient or narrow domain datasets can possibly reduce the performance of the pretrained model on other datasets (a concept referred to as “catastrophic forgetting”) [56]. However, other researchers have noted the contrary [57]. In any case, it would be important to verify the ASR models fine-tuned with investigative interview data improves performance across possibly underrepresented dialects, demographics, and criminal domains.

Other challenges would apply to any given provider and deployer and their datasets, such as the fact that transcription conventions vary from dataset to dataset, and domain to domain [51], and that different ASR solutions may also require different degrees of verbatim transcription for training. Models like Wav2Vec2 will require a more verbatim transcription for training, whereas sequence-to-sequence models like Whisper only need what the speaker intended to say.

#### 4.2.2 Dataset properties

According to Art.10 (3) datasets must be relevant, representative, complete and to the best extent free of errors in relation to the purpose of automated speech-to-text transcription and with specific properties for ASR systems.

**Relevance, Completeness, and Error free**

As there are no specific definitions distinguishing relevance and suitability, we assume they refer to the same activity, that the dataset is capable of training or testing an AI system such that it can accomplish its given purpose. Thus, see the above subsection for a discussion regarding dataset relevance.

“Completeness” is also not explicitly defined in AIA, but presumably it is intended to capture all ideally necessary factors in speech recognition, such as a sufficiently large and relevant vocabulary, various speech rates, and other speech variance such as emotional state [51]. Veale and Zuiderveen Borgesius [58] state that fully satisfying completeness and being free of errors are “steep” tasks. In order to satisfy completeness, it would seem to imply that a large amount of audio-text data should be collected that attempts to cover as many of these expected variables as possible. While foundation models and their datasets may be sufficiently complete for their purpose (such as transcribing non-domain specific speech), the assumption is that any fine-tuned models will be “more complete” for their given purpose (whether for transcribing regional dialects or specific domains). Ultimately identifying when a dataset is complete is in and of itself a challenge, especially when abiding by the GDPR data minimisation principle. Comparisons with similar research may provide guidance on how much fine-tuning data is needed to reach certain improvements, and larger and more diverse testing sets will always provide more confidence in how an AI system will work generally.

“Representative” appears to describe that the intended demographics of the AI system are appropriately present in the datasets, and many of the principles which apply to completeness apply here as well. As mentioned previously, low-resource languages may struggle to have enough training or testing data regarding their demographics. The language itself may be underrepresented as a possible resource, but also more niche elements such as dialects, foreign accents, a balance between the sexes, or between the young and the elderly.

Being free of error means that the reference transcripts for any audio samples should be transcribed *sufficiently* correctly. Being completely free of error is unlikely for a number of reasons. The first is that transcription conventions vary between datasets, domains, and models, and transcribers may simply transcribe the same audio differently. And like machines, human transcribers are also prone to making mistakes, where a study has shown that human transcription for a clean and read dataset had a word error rate of 5.8% [59]. Another issue is that it appears that pseudo-supervised training is effective enough to improve models such as Whisper [49], [52], which means that transcriptions made by machines for datasets are “good enough”.

For investigative interviews, the quality of at least the testing data should be high given the requirement of verbatim transcription. For mitigating errors when creating datasets, ideally multiple transcribers should be used (whether or not supported by ASR), and any identified errors in the dataset should be updated over time [49]. However, resources for such careful annotation are unlikely available for the police.

**Application-area Specificity**

While there have been attempts to generalise models well to unseen datasets [9], [49], it worth noting that models tested on data they were not trained on tend to perform less well than models that are trained and tested on the same dataset (where the testing data is held out) [60]. Thus, it is ideal that any ASR system used for investigative interviews are at least tested on data from within that domain in terms of expected vocabulary and audio content. For example, important for the study of application of ASR systems to Norwegian investigative interviews would be voice recordings of children and other vulnerable persons, both of which are sensitive and would require full transcriptions.

### 4.3 Transparency, technical documentation, and record keeping

Transparency is an overarching principle in the AIA. Technical documentation ensures accountability (Art.11) and record keeping is related to the possibility to audit processes (Art.12) - both of which aim to make the system more transparent.

#### 4.3.1 Transparency

In order for an AI system to be considered transparent, Art.13 requires deployers *(i)* to be able to interpret its output and *(ii)* to be provided with sufficient information about the components that impact the AI system performance. The AIA does not define interpretability of AI system output, while in techno-legal context the term is used interchangeably with explainability (XAI) [61]. There is an increasing amount of research in defining the criteria [62] and the methods and metrics [63] for interpretable AI. AI systems may have different degrees of interpretability as interpretations are context and domain specific, but essentially it means that “it is not necessarily required to provide an interpretable representation of a mathematical model, but most importantly to provide a train of thought that can make the decision meaningful for a user” [62]. In this sense, AI systems that are used for evidence analysis and reliability evaluation need to be more transparent in terms of the dataset and feature engineering, preprocessing and modelling, and output interpretation, than a system for interview transcription.

Generally, in machine learning there are two primary methods to assess model interpretability: some where “interpretability is achieved through constraints imposed on the complexity of the ML model (intrinsic)” or others where it is achieved “by applying methods that analyse the model after training (post hoc)” [63]. Current ASR models predominantly fall in the second category. Consequently, although an ASR system might not be fully explainable, its output is interpretable and legally this should be sufficient for a low-risk AI system.

**Fig. 1:**
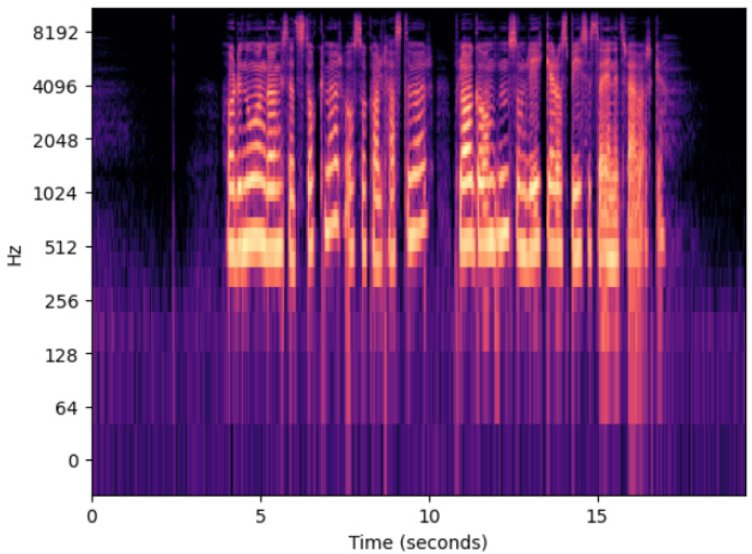
Example log Mel Spectrogram derived from the first audio file from the NB Tale Module 1 dataset, which can be the features used for speaker recognition tasks. The X-axis denotes time, whereas the Y-axis denotes frequencies of the audio file on a logarithmic Mel scale (which is more akin to how humans perceive sound) over time. Brighter colours denote the amplitude of those frequencies over time.

Generic black box approaches such as LIME [64] can be used to isolate which input features contribute to a classifier’s decision (such as specific sets of pixels for image classification or words for text classification). But unlike image or text classification systems, ASR systems for speech-to-text do not have an immediately intuitive method for post-hoc explainability [65], [66]. Features in ASR systems are typically values derived from short frames (e.g., 25 ms) of sequenced audio, such as time series amplitudes of a frame of a raw .wav file, the log Mel spectrum of a series of audio frames (see Fig. 1), or the Mel Frequency Cepstral Coefficients (MFCC) of a series of audio frames [13].

Despite the challenges in interpretability, past research has attempted to apply explainable AI to ASR. Black box approaches include identifying which portions of a spectrogram contribute to letter outputs [66], and “zeroing out” parts of a spectrogram to see which features influence predictions [67], [68], [69]. White box approaches to ASR have been researched as well, wherein researchers have examined which layers of an end-to-end neural network contribute to encoding phonemes or graphemes [65], [70].

Interpretability approaches for ASR and their errors are also being researched by calculating confidence levels for word transcriptions [71], [72]. Furthermore, it has been shown that word level confidence scores can be obtained from systems like Wav2Vec2^7^ and Whisper.^8^ In all likelihood, these approaches would be most practical for explaining model decisions for law enforcement.

#### 4.3.2 Technical documentation

Art. 11 AIA requires extensive documentation of AI models in accordance to Annex IV including, general description of the AI system, the elements of the AI system and of the process for its development, the validation and testing procedures used, about the monitoring, functioning and control of the AI system, and the risk management system. Technical documentation challenges for ASR models exist in relation to documenting suitable performance measures as well as testing procedures.

In terms of appropriateness of performance metrics requirement in Annex IV (4), a challenge within the field of ASR is that the word error rate (WER) is a flawed metric, despite its universal use.

The WER does not distinguish between the weight or importance of the errors, so a minor spelling error in a word is counted just is much as the insertion of a word that completely alters the context of what was being said. Nor does the average WER convey the shape of the performance distribution over all tested speakers [51]. There are examples in the medical realm that report ASR WER results that also include the median, standard deviation, and the range [73], where knowledge of the standard deviation can illustrate if an improvement or distinction between populations is actually statistically significant. Even considering its flaws, the WER is considered the best metric. Past work has suggested a number of different approaches to compare the differences between the reference and machine produced transcription. One such suggestion is to compare how closely texts are semantically utilising Natural Language Processing approaches [73]. Older suggestions consider the Match Error Rate and the Word Information Lost [74], though they are less often used. The Character Error Rate is still quite relevant for logographic writing systems such as Chinese.

What is often missing in ASR studies that would be relevant for informing performance in transcribing investigative interviews are the timings of how long on average transcriptions took. Aksënova et al. [51] suggest presenting a ratio between the “wall clock time” (hardware independent) taken to transcribe the audio by the ASR system to the length of the audio itself, and also suggest to test systems on out-of-domain data to weed out “hallucinations” (see Section 4.5.1).

Art. 12 AIA requires record-keeping to ensure accountability and transparency for the lifetime of the system. In ASR models logging can be assured in terms of times of use. Data retention policy should define strict limits for the storage of initially transcribed audio and audio/text pairs. For verification purposes, it will be necessary to store such initial transcriptions alongside the final corrected draft in the police system.

### 4.4 Human oversight

Art. 14 AIA requires built-in or user-implemented measures to allow a person to correctly interpret the high-risk AI system’s output. The requirement of human oversight is often seen of limited use since humans “will often not be able to second-guess the validity of the [AI] system’s outputs, except in limited cases where human intuition may detect [only] obvious failures or outliers” [75]. Thus, a human in the loop should be implemented as a verification procedure where the examiner can verify the content of the transcript, correct post-processing errors ensuring human supervision of the transcripts from AI output. It also gives the opportunity of the speaker or a representative to object inaccuracies. Although, AIA does not include rights for subjects of AI systems, such rights are guaranteed under data protection law and the fair trial principle.

However, as previously argued human transcribers cannot only replicate bias in the system, but even create it post factum e.g., insert text transformations that are biased or benefit the prosecution. Human transcriber bias is difficult to measure as it may vary from case to case and may be triggered on individual basis e.g. sympathy or dislike to the suspect, prejudice against certain behaviour or attitude. An ASR model may still preserve bias but it can be consistently detected, tested, and mitigated. However, there are insufficient studies to compare ASR versus human biases and their effects on the overall quality of verbal evidence. It is also important if the human transcriber will have an opportunity to correct inaccuracy in the transcription in real-time (in a transcription streaming scenario) or after the interview is finished. It remains unclear from the AIA text who is competent to conduct this oversight on ASR systems, what competence and training they should have and how human oversight audits should be performed.

### 4.5 Accuracy, robustness, and cybersecurity challenges

Further required, is that AI systems must have an appropriate level of accuracy, robustness, and security throughout their life cycle (Art. 15 AIA). As those are very distinct subjects regarding the performance of ASR systems, we examine them further separately.

#### 4.5.1 Accuracy

In the field of ASR, performance is clearly seeing improvements over time, but what is noted as sufficient accuracy depends on many different factors. For instance, Microsoft suggests that a WER between 5%–10% is “good quality” and ”ready to use”, whereas 20% is considered to be “acceptable” and 30%+ is considerably poor quality and requires “customisation and training” [76]. Gaur et al. [77] also found that at a 30% error rate, correcting a transcription produced by an ASR system is no faster than a human performed transcription. However, accuracy depends on the domain and the possible severity of risks involved. A risk analysis study considering the use of ASR for air traffic control in Chinese and found a character error rate of 3% intolerable where a majority of errors was due to incorrectly transcribing numbers given their high base rate [78].

At this time it is difficult to say how well ASR systems perform on investigative interview recordings due to few previous studies and in-domain data (and other than some internal positive experiences). Using mock interview data, the study by Harrington [15] found the WER for interviews varied between roughly 14%–34%, where this accounts for both accents and the ASR system used in a studio environment. The authors, however, do not comment directly on the influence of the domain of the data on performance.

To get a better perspective on how ASR might perform within the domain of investigative interviews, we can look at ASR performance in audio contexts that are similar to an interview. Considering typical interviews that take place in a controlled environment, it can be considered that the fidelity of the recordings should typically be high with minimal background noise, the speech somewhat conversational and mostly spontaneous, and there are at least two people speaking, the interviewer and the interviewee.

It is well known that the WER is considerably lower for read or prepared speech (5% WER possible) than it is for spontaneous speech (less than 10% is exceptional) [79], though with recent improvements in ASR technology the gap between these categories is appearing to become narrower. Multi-speaker environments increases the error rate due to possibly overlapping of speech [80].

Assessing an ASR system’s performance on investigative interviews may also differ from typical testing procedures of ASR models, since oftentimes common ASR benchmarks consider the average performance across *utterances* of a dataset (a stretch of continuous speech), where the duration of utterances is typically less than 30 s [81]. Thus in practice, it is not only the ASR model which needs to be considered, but also the influence of the ASR system which breaks up longer audio files into smaller pieces before the ASR model is applied. OpenAI’s Whisper and Huggingface’s Transformers pipeline both apply “chunking” to longer audio files, wherein audio files are partitioned into smaller overlapping segments such that they can be ingested by the models.

Specific demographics may also have a statistically significant influence on ASR output. When comparing performance between men and women, generally it is found that men tend to perform better than women likely since they are more present in training datasets (though the gap is typically small) [82], [83]. Age also appears to considerably affect ASR systems, as studies have shown decreasing WER for speakers of 65 and older [84], and significantly worse performance for child speech [57], [85], [86]. A study using a Wav2Vec2 model fine-tuned on adult speech obtained a WER of 35.3% on the speech of Swedish children [87]. However, a more recent study has shown the ability to obtain greatly improved ASR performance on native and non-native English child speech when fine-tuning Whisper Version 2 on an additional 1 to 50 h of speech data [57]. Their results showed that most accents achieved <12% WER, except for Chinese (14.31% WER) and German (34.26% WER). The authors also note that the performance of the models on general datasets were not reduced (no observed catastrophic forgetting), but the tested corpus may have been too easy as it was read English. For dialects and accents in general, many studies have established that there is a disparity between native or more popular accents than smaller regional dialects or foreign accents [42], [43], [88], [89], [90], [91].

A practical matter for interviews which is typically treated separately from the ASR task, is assessing the performance of speaker diarisation [92] (that is, segmentation between speakers). Ultimately, such designations are necessary to include in an interview transcription. While discussing this in depth is out of scope for our current work, the top performing diarisation methods have achieved a 4.3% error rate and less on the VoxCeleb Speaker Recognition challenges from 2021-2023 [93].

#### 4.5.2 Robustness against errors

When speaking of robustness of ASR systems, Art. 15 AIA and recitals 75–76 refer to two broad categories: resiliency “against risks connected to the limitations of the systems (e.g. errors, faults, inconsistencies, unexpected situations)” and then resiliency against “malicious actions that may compromise security of the AI system” which may influence individuals‘ fundamental rights or produce biased outputs.

In terms of common errors and inconsistencies for tests on homogeneous datasets or groups, this can be partially identified by not only using the average word error rate, but another measure which captures the variance of the error rates as well. An average word error rate may be deemed acceptable, but a low average WER does not necessarily imply high consistency in performance. Thus, to indicate the overall distribution of the errors for a given test, some measure of variance should also be shown in addition to the average WER.

Another challenge worth considering for robustness of AI systems is the rate of “hallucinations”. Hallucinations are a special category of error where text output by an ASR system (or other generative model) is considered to be nonsensical or unfaithful to the original text [94], [95]. The dangers of such errors are that as opposed to an ASR system producing text with a few words off or an incorrect spelling, is that the transcription may now be inserting something that was not even remotely close to what was being said. A particularly relevant study conducted by Koenecke et al. [39] found that when testing the Whisper API in 2023 that 1% of produced transcriptions contained hallucinated sentences or phrases. However, when analysing the content of the hallucinations they found that 19% of them included a “perpetuation of violence”, and 13% of them made up names or facts. The authors of the study tested systems other than Whisper but concluded that “hallucinations to currently be an OpenAI-specific concern” [39]. Research has also attempted to develop methods to automatically identify hallucinated text [96].

More recently, an increasing amount of research has focused on issue or robustness in terms of domain specificity of training and testing datasets. As stated before, a model which was trained and tested on a specific dataset, is not guaranteed to work well on a different dataset [60]. Research into more robust models have shown training on a large variety of data improves the robustness of ASR systems for datasets that was not used for a model’s training [9], [49]. Increased robustness is important in scenarios with expected high signal-to-noise ratios, as exemplified by Field et al. [97], where it was shown that fine-tuning ASR models on in-domain data for body camera audio during traffic stops performs well for white and black American officers, but poorly for the drivers, and instances where multiple officers had overlapping speech.

#### 4.5.3 Cybersecurity robustness

Like other machine learning approaches, ASR systems are susceptible to adversarial machine learning. In particular, such attacks have the goal of forcing an ASR system to produce an incorrect or targeted transcript for some given audio with the introduction of adversarial noise [98].

Carlini and Wagner [99] have shown, given the attacker has access to a model and its parameters, that it is possible to conduct a targeted adversarial attack on any given audio file x such that by inserting perturbations on the audio to create $x^{\prime }$ it is possible to produce any desired transcription output. Such attacks have been shown to be possible on Whisper as well [100].

Olivier and Raj [101] have also studied how well adversarial attacks can transfer between models, given a white box assumption, and have found that while past hybrid ASR models have been robust against transferable targeted adversarial examples, the different versions and architectures of the Wav2Vec2 models are not. Attack scenarios in which attackers have no knowledge of a model or its parameters (a black box assumption) typically require sending a large number of queries to an ASR API to essentially randomly determine if a given file audio perturbation will succeed as an adversarial example [102]. Suggestions have been given that possible mitigation to such attacks is to increase the robustness of ASR models in terms of performance [99], such as by conducting adversarial training [102].

Another common form of attack against machine learning systems is that of dataset poisoning, where a machine learning model is provided with false or misleading data such that the model is maliciously trained, thereby producing outputs which may benefit an attacker. While more common in the image processing domain, data poisoning for ASR systems is deemed far more complex due to the time-series nature of speech-to-text data. However, a recent study by Aghakhani et al. [98] showed that targeted data poisoning attacks are possible when pre-training and fine-tuning traditional hybrid ASR models, but even work on some end-to-end models as well. Mitigations against poisoning attacks include verifying the training dataset and attempts for automated detection of poisoning [103].

The previous research indicates a need for confidentiality and integrity being applied to a model, system, or dataset. Furthermore, such data or systems should have restricted access to the internet, especially in cases where real interview data is being used.

This overall voluntary assessment for compliance with the high-risk requirements in AIA shows that risks to privacy, fair trial, discrimination, and security exist even in systems (like ASR models for interviews) that do not fall under the high-risk categories. In general, the dualistic approach in AIA of either high-risk or no-risk system for law enforcement as well as the limited, list-like approach to define prohibited practices or high-risk systems is criticised as too simplistic as it frames ‘complex problems by means of dichotomies’ [104]. Rather, a more tailored approach is needed where criminal procedure questions arising from the new technology are systematically evaluated and weighted for new safeguards.

## 5 Discussion

Table A.1 (found in the Appendix) presents an overview of the identified challenges and initial suggestions for a voluntary code of conduct for ASR systems in law enforcement work. We summarise and critically examine the AIA-based criteria for the assessment of ASR systems for investigative interviews. This section further answers the research questions by highlighting the uncertainties and solutions which cross different AIA provisions, and emphasises that the AIA may be too vague and broad to address criminal procedure specifics.

### 5.1 What is the legal classification of ASR systems for investigative interviews and the related LEA obligations according to the AIA?

The first relevant question for law enforcement when introducing an ASR system into their workflow is to determine the risk level of the AI system. In Section 3.1 we provided an argument that ASR for the purpose of transcribing investigative interviews is not high risk according to the AIA as the ASR system is not used for speaker identification and categorisation or assessment of reliability of verbal evidence.

Our assessment concluded that ASR models does not fall within the definition of general-purpose AI (GPAI), thereby not requiring any compliance with the specific transparency regime for GPAI. However the definition of GPAI raises several ambiguities in relation to foundation models which require clarification of the concepts of self-supervision to include pseudo- and weakly-supervised models and developing of a more robust criteria to assess generality and task of a model. As examined in 3.1.1 generality does not necessarily result in capabilities to perform wide variety of tasks, as pretraining and fine tuning can significantly reduce the capabilities of a general AI model. Rather than focusing only on amount of parameters used in a model as a measure to generality, clarifying the concepts in the context of pre-training methods will be crucial for the correct interpretation of AIA in practice. Even as minimal risk and non-GPAI systems, it is recommended that ASR for investigative interviews are subject to voluntary compliance with the AIA high-risk requirements due to the possible impact of automation on criminal procedure.

The second important question for law enforcement is to determine their responsibilities as a provider or deployer according to AIA. We argued, that when foundation models are used in AI systems for law enforcement purposes, the AIA is not specific enough to address issues of fine-tuning and re-purposing of ASR models which might result in power imbalances between commercial or GPAI providers and law enforcement as a downstream provider or deployer. The tendency of LEA to use all-in-one tools, that they them-self have not tested, creates technology dependencies and reliability issues in LEA work. Further challenges arise on instances where the police want to fine-tune and validate commercial AI models with their own in-domain data (such as real investigative interviews). In such circumstances, voluntary compliance for high-risk AI systems is difficult to be met, as they are unlikely to have the personnel, data, or resources to satisfy the testing and data requirements.

### 5.2 What are the challenges and mitigation strategies that law enforcement should consider in developing a voluntary code of conduct for ASR systems transcribing investigative interviews according to Art. 95 AIA?

The assessment of ASR systems for investigative interviews shows that several, non-trivial technical and legal challenges must be addressed in practice in order to ensure compliance with AIA.

Risks to privacy, fair trial, non-discrimination, and security exist even in systems (like ASR models for interviews) that do not fall under the high-risk categories. Moreover, risk mitigation in such cases is related not only to safeguards in the AI system itself (Art. 9 AIA), but rather evaluation if the existing rights and obligations in the concrete criminal procedure are sufficient in the context of their automation.

In particular, risks to fair trial are important to take into account, as some ASR models such as Whisper have the possibility to hallucinate content and potentially add false or incriminating elements to a transcription [39]. This is perhaps an ASR model’s most concerning attribute regarding their robustness. Other than their typical transcription errors, this justifies the need for human oversight by ensuring a law enforcement individual corrects the draft written by the ASR system, and allows the interviewee the opportunity to object to the automatic transcription and corrections (Art. 14). Although ASR systems are low risk systems according to AIA, they still process personal and sensitive data and must be in compliance with LED. Regarding fairness, an ASR system may contribute to criminal procedure since they cannot hold superficial biases against a user (such as a presumption of guilt or outright bigotry), despite the fact that ASR systems demonstrate statistically significant differing results for different demographics [42], [83], [84], [91]. In terms of bias mitigation for non-discrimination there are sufficient testing techniques to address ASR issues with different demographics [43], [44], [45]. Required are (demographically) representative datasets for the training and testing.

Satisfying data governance (Art. 10) challenges for any party will be challenging, as the expectations for datasets to be relevant, complete, and free of error contrasts with how recent ASR datasets are made. Modern pre-training data may be sufficiently complete, but in the case of Whisper unlikely free of error since the data may not be validated by humans (in the case of weak-supervision) or the data may also be produced in automated fashions to support pseudo-supervised learning. It remains to be seen if the shift from data volumes to domain-specific, curated datasets as envisioned by the legislator will be achieved in practice considering the significant transaction costs for dataset engineering. Domain-specific datasets, may also be scarce due to their sensitivity (such as investigative interviews). Thus, even if the datasets are carefully transcribed and annotated, they are likely to be incomplete simply due to the fact that such data is scarce. We note that their scarcity is amplified by the need to comply with GDPR strict control over sensitive data and the focus of data minimisation (a factor which directly contrasts the need for completeness). Thus, we tend to agree with previous statements that meeting the need of data to be free of error and complete is perhaps a technically impossible task [58]. Despite these challenges, it is important that LEAs can gather a sufficient amount of data at least for testing their ASR systems, as a system compared against one benchmark is not guaranteed to be perform similarly in a different specific domain [60] (such as investigative interviews). For the purpose of accountability and record keeping of the use of an ASR system (Art. 12), data regarding the usage of the ASR system (who and when), the audio, initial ASR transcription, and final transcription draft reviewed by LEA and approved by the interviewee should be stored. This is to ensure that any opposition to the final interview can be checked against the original material.

Another challenge that we highlight is that the internals ASR models (Art. 13), like other deep learning models, are not fully explainable. Complicating this matter further is the fact the features from audio data are not so readily interpretable as other kinds of data (such as images or text), so typical explainable AI methods tend to be less useful. However, models can still produce word or chunk level confidence scores, and this should be sufficient for a low-risk ASR systems compliance with AIA.

The *accuracy* (Art. 15) of ASR systems, while tending to improve [79], still have a number of challenges law enforcement should be aware of. A specific demographic ASR models have historically shown to struggle with is the speech of children [57], [85], [86], an important use case for law enforcement transcription. Furthermore, while there are expectations of what is a “good” error rate, different domains may find particular word error rates or kinds of errors intolerable for their use [76], [78], and no studies have shown what kinds of errors or error rates are tolerable for investigative interview transcription. Furthermore, LEA needs to be aware that the WER is a flawed metric all types of errors are weighted equally, but the best we currently have (Art. 11).

The security of ASR models, audio data, and transcripts for law enforcement (Art. 15) is also an important challenge to address, as methods exist to manipulate the output of an ASR system to benefit an attacker. Targeted adversarial attacks exist such that a malicious actor may manipulate an audio file to produce a transcript benefiting the attacker, while the audio appears benign [99], [100]. Data poisoning attacks also exist in which bad audio text pairs are added to the dataset to diminish or force particular transcription outputs [98]. Possible mitigation’s exist to increase robustness against attacks, such as adversarial training [26], or data poisoning detection approaches [103].

### 5.3 Limitations of the study

AI has become a fast-moving target, where new ASR models tend to significantly outperform previous state-of-the-art models in only a matter of months. We observe this is a consequence of foundation models becoming larger, where they use significantly more training data and compute time. Thus, considerations of model performance and how they might perform in an investigative interview will require near constant updating.

Our study has also only performed the full analysis of some of the most popular open-source ASR models in the context of AIA compliance. This was to limit the scope of the study, but also simply because we have neither the insight or access to close-sourced commercial models. Even so, the related studies often show that Whisper tends to outperform commercial models, while we discussed the issues with closed-source models for law enforcement purposes.

Lastly, the provided code of conduct suggestions are considered initial advice to LEA and other parties required to comply with the AIA, and is not considered to be a full “compliance checklist”.

## 6 Conclusion

Our study has argued that Automatic Speech Recognition (ASR) for transcribing police investigative interviews would be considered a minimal risk AI system, which does not include GPAI model use in the view of the recently adopted AI Act. The paper has also provided groundings of why voluntary compliance to the AI Act’s high-risk requirements, even when using open-source ASR models would be highly recommended but also challenging. In doing so, we have provided an initial analysis for the development of a voluntary code of conduct (see Table A.1). It highlights challenges and mitigation strategies for law enforcement to consider when adopting ASR solutions. Due to the current paradigm of using foundation models, this code of conduct may also be used as a basis for the evaluation of other AI systems.

The paper has also demonstrated that AIA general requirements such as model generality, task, self-supervision, or provider must be clarified in the law enforcement domain. It is argued, that even systems that are not classified as high-risk (such as ASR) in criminal procedure settings might have an impact on the fair and equal treatment of suspects, defendants, victims, and witnesses. While the approach of AIA to ensure accountability regime for AI systems is very beneficial, its successful adoption by law enforcement will depend also on evaluation if the rights and obligations in the concrete criminal procedure are still preserved in the context of its automation and if new rights and obligations should be introduced in order to ensure that individuals will not bare any negative consequences.

In terms of future work, it may be useful for similar interdisciplinary analyses to be performed for various AI applications for specific use cases as it allows to contextualise the new AIA in relation to practical solutions.

**Table A.1:** Suggestions for a voluntary code of conduct for ASR systems for investigative interviews.

AI act requirements	Challenges and suggestions for ASR systems for investigative interviews
Risk-management system Art. 9	**Challenges:** • Risk-management in ASR systems is not only related to safeguards in the AI system, but also to evaluation if the existing rights and obligations in the criminal procedure for police interviews are sufficient in the context of their automation. • The legal regime for providers and deployers does not consider public–private cooperation and creates dependencies for law enforcement on the initial AI providers in case of low-risk tasks, which in the domain of criminal procedure are best to be avoided. **Suggestions:** • Determine who is legally responsible for AIA compliance (providers and deployers). • As a provider of repurposed commercial AI model, law enforcement can account only for the fine-tuning of the model and domain specific data testing.

Data and data governance Art. 10	**Challenges:** • It is unlikely that pre-training or fine-tuning datasets will fully meet requirements of being complete, relevant, or free of error. • Transcription standards for people and datasets, as well as different ASR models, are inconsistent. **Suggestions:** • Law Enforcement should test the ASR systems on their in-domain investigative interview data that is deemed sufficiently relevant and complete. Any fine-tuning of models should be sufficiently large to avoid catastrophic forgetting.

Technical documentation Art. 11	**Challenges:** • Word Error Rate is universally used to measure performance but it is a flawed metric. • Semantic similarity between transcriptions is still an open problem. **Suggestions:** • Testing should also report average transcriptions time.

Record-keeping Art. 12	**Suggestions:** • Automatic logging of chain of custody details for each interview (including date and time, participants, human oversight, and requested corrections by each party). • Data retention of initially transcribed audio and audio/text pairs is necessary in addition to the final draft.

Transparency information Art. 13	**Challenges:** • Predominantly post hoc interpretability. **Suggestions:** • Use of word level or chunk level confidence scores.

Human oversight Art. 14	**Challenges:** • Lack of research on ASR versus human biases and their effects on the overall quality of verbal evidence. **Suggestions:** • Use ASR to produce drafts that are ultimately checked, verified, and updated by a human.

Accuracy Art. 15	**Challenges:** • Different domains have different requirements of what is an acceptable Word Error Rate. • Standard benchmark settings may be too simple to infer performance of real investigative settings. • Disparity between popular accents and regional (and foreign) dialects **Suggestions:** • In the context of testing procedure: full audio files should be evaluated, not only short utterances.

Robustness Art. 15	**Challenges:** • Hallucination risks, especially those which make up text referring to false statements or information. • ASR systems are prone to noise, and less popular voice types (children, regional dialects, etc.) **Suggestions:** • Understand what is minimum error threshold for investigative interviews, and the types of errors which may spoil a transcript. • Also measure variance of the error rates.

Cybersecurity Art. 15	**Suggestions:** • Need for more automated poisoning detection and verification of training datasets as well as adversarial training research to counter targeted attacks, and examine effect on general performance

Specifically for investigative interviews, more studies need to be done exploring the performance of state-of-the-art ASR models on real investigative interview data. This is needed as it is unlikely that pre-trained foundation models were trained on such data, and thus may not perform as well as other more general data benchmarks may indicate. Furthermore, studies demonstrating the effect of fine-tuning of such models on investigative interview data would be of interest to determine possible system improvements given a potentially scarce data resource. Such models should be tested across various types of criminal cases (a child abuse case versus an economic crime case) to determine if the models suffer from catastrophic forgetting. Other practical technical studies should include the determining the benefits of how much faster transcription of interview can be when using ASR systems and other supporting technology such as speaker diarization, and determining the error thresholds of when an automatic transcription may be deemed sufficiently useful.

## CRediT authorship contribution statement

**Radina Stoykova:** Writing – review & editing, Writing – original draft, Validation, Methodology, Investigation, Conceptualization. **Kyle Porter:** Writing – review & editing, Writing – original draft, Validation, Project administration, Methodology, Investigation, Funding acquisition, Conceptualization. **Thomas Beka:** Writing – review & editing, Conceptualization.

## Declaration of competing interest

The authors declare the following financial interests/personal relationships which may be considered as potential competing interests: Thomas Beka reports a relationship with Norwegian Police IT-unit that includes: employment. If there are other authors, they declare that they have no known competing financial interests or personal relationships that could have appeared to influence the work reported in this paper.

## Appendix Suggestions for voluntary code of conduct

See Table A.1.

## Footnotes

1 According to a recent anecdotal survey within the police.

2 https://huggingface.co/openai/whisper-large-v3.

3 Bommasani et al. [23] have called Wav2Vec2 a foundation model for ASR.

4 Norwegian Regulations on the system of the public prosecution (Påtaleinstruksen), Section 8-14, https://lovdata.no/dokument/SF/forskrift/1985-06-28-1679/.

5 https://github.com/snakers4/silero-models.

6 For Norwegian, the Norwegian National Library has done excellent work in developing and publicly releasing dozens of such datasets and downstream models [54], [55].

7 https://discuss.huggingface.co/t/confidence-scores-self-training-for-wav2vec2-ctc-models/17050.

8 https://github.com/openai/whisper/discussions/284.
